# Regulation of BLM Nucleolar Localization

**DOI:** 10.3390/genes7090069

**Published:** 2016-09-21

**Authors:** Larissa Tangeman, Michael A. McIlhatton, Patrick Grierson, Joanna Groden, Samir Acharya

**Affiliations:** 1 Department of Cancer Biology and Genetics, College of Medicine, The Ohio State University, Columbus, OH 43210, USA; larissa.tangeman@osumc.edu (L.T.); michael.mcilhatton@osumc.edu (M.A.M.); 2 Divisions of Hematology and Medical Oncology, Department of Internal Medicine, Washington University School of Medicine, St. Louis, MO 63110, USA; grierson@wustl.edu

**Keywords:** Bloom’s syndrome, rRNA transcription, BLM, topoisomerase I, growth defects, nucleolar localization

## Abstract

Defects in coordinated ribosomal RNA (rRNA) transcription in the nucleolus cause cellular and organismal growth deficiencies. Bloom’s syndrome, an autosomal recessive human disorder caused by mutated recQ-like helicase BLM, presents with growth defects suggestive of underlying defects in rRNA transcription. Our previous studies showed that BLM facilitates rRNA transcription and interacts with RNA polymerase I and topoisomerase I (TOP1) in the nucleolus. The mechanisms regulating localization of BLM to the nucleolus are unknown. In this study, we identify the TOP1-interaction region of BLM by co-immunoprecipitation of in vitro transcribed and translated BLM segments and show that this region includes the highly conserved nuclear localization sequence (NLS) of BLM. Biochemical and nucleolar co-localization studies using site-specific mutants show that two serines within the NLS (S1342 and S1345) are critical for nucleolar localization of BLM but do not affect the functional interaction of BLM with TOP1. Mutagenesis of both serines to aspartic acid (phospho-mimetic), but not alanine (phospho-dead), results in approximately 80% reduction in nucleolar localization of BLM while retaining the biochemical functions and nuclear localization of BLM. Our studies suggest a role for this region in regulating nucleolar localization of BLM via modification of the two serines within the NLS.

## 1. Introduction

Ribosomal RNA (rRNA) transcription plays a central role in ribosome biogenesis with rRNA an integral part of ribosomal subunits [1,2,3,4,5,6]. The genes encoding rRNA (*rDNA*) are found in multiple copies organized into tandem repeats; human cells contain more than 400 *rDNA* clusters of repeats, each spanning 35 kb on the p-arms of the acrocentric chromosomes 13, 14, 15, 21 and 22. *rDNA* loci are characterized by clusters of GC-repeats capable of forming aberrant structures during replication and transcription, and undergoing intra- and inter-chromosomal recombination. Transcription of *rDNA* genes is facilitated primarily by the RNA polymerase I complex in the nucleolus, with the 18S, 5.8S and 28S rRNAs transcribed as a single pre-rRNA transcript that is subsequently processed prior to the assembly of ribosomes [2,7,8,9]. The eukaryotic ribosome consists of a small (40S) and a large (60S) subunit: the 40S subunit includes 18S rRNA and 33 ribosomal proteins; the 60S subunit includes 5S rRNA, 28S rRNA, 5.8S rRNA and 46 ribosomal proteins [6,7,8,9,10]. Ribosome biosynthesis requires the coordinated activity of three RNA polymerases, Pol I (for 5.8S rRNA, 18S rRNA and 28S rRNA), Pol II (for mRNA encoding ribosomal proteins) and Pol III (for 5S rRNA), to synthesize rRNA and ribosomal proteins in the required stoichiometric amounts for assembly into ribosomes [6,10,11].

Ribosome biogenesis is critical for protein translation, cellular growth and proliferation. The group of disorders termed ribosomopathies, characterized by defects in the ribosome biogenesis pathway and including mutations in RNA polymerase I transcription complex components, results in severe growth impairment [4,10,12,13]. Furthermore, dysregulation of *rDNA* metabolism is associated with nucleolar dysfunction, cellular and organismal growth defects, and cellular transformation; it is often suggested as a primary cause of cellular senescence [3,9,10,14,15,16,17,18]. Dysregulation of *rDNA* metabolism correlates with *rDNA* repeat instability and compromised DNA damage responses throughout the genome.

Bloom’s syndrome (BS), an autosomal recessive human disorder caused by mutations of the recQ-like helicase BLM, is associated with several characteristics of nucleolar dysfunction [19,20,21,22,23,24]. Individuals with BS display intra-uterine growth retardation and proportional dwarfism that persists throughout life. BS is an extreme case of a ribosomopathy that presents the apparent paradox of growth retardation, a consequence of hypo-activity of the nucleolus, and a high predisposition to cancer, often associated with hyperactivity of the nucleolus [13]. The intersection of a growth retardation phenotype and cancer induction is intriguing. Most, but not all, ribosomopathies present with hematopoietic defects; however, the mechanisms underlying these defects are unclear. It is thought that ribosome deficiency results in p53-induced apoptosis in rapidly proliferating hematopoietic precursor cells, resulting in the observed clinical phenotypes. Differences in the clinical phenotypes among various ribosomopathies could be due to differential protein expression and thresholds for p53 activation among various tissues [13,25]. Treacher Collins syndrome, caused by mutations in the treacle protein, presents with craniofacial defects similar to the classic ribosomopathy Diamond-Blackfan anemia, but without hematologic abnormalities. A mouse model of Treacher Collins syndrome recapitulates the ribosome deficiency and craniofacial abnormalities [26]; these craniofacial abnormalities are rescued by inhibition of p53 [27]. Hematopoietic defects are not generally found in those affected by BS, although an embryonic-lethal *Blm* knockout mouse model results in severe anemia during embryogenesis [28]. Cells without BLM display impaired growth in tissue culture and are characterized by elevated *rDNA* repeat instability, inter- and intra-chromosomal recombination, recombination of acrocentric chromosome arms where *rDNA* is found, chromosomal breakage and telomeric associations [19,20,22,29,30,31]. The mechanisms underlying the clinical growth defects of BS remain unclear.

Several studies point to a nucleolar role of BLM in *rDNA* metabolism and stability. BLM localizes to the nucleolus via the C-terminus and to promyelocytic leukemia (PML) nuclear bodies, implicated in DNA repair mechanisms, via the N-terminus [32,33]. BLM functions as an ATP-dependent 3′–5′ helicase and aids in the resolution of unusual DNA structures associated with replication stress and recombination especially for the GC-rich DNA sequences found in *rDNA* and at telomeres. BLM directly associates with *rDNA* sequences, specifically with the *18S*-coding and *Alu*-repeat regions; its localization correlates with genomic stability [31,33]. BLM deficiency results in hyper-recombination within *rDNA* [34]: BS cells display reduced overall *rDNA* repeat numbers in comparison to wild-type cells. Furthermore, our recent studies demonstrate a role for BLM in facilitating *rDNA* transcription in the nucleolus [35,36]. BLM physically interacts with several RNA polymerase I complex proteins, including DNA topoisomerase I (TOP1), and accelerates *rDNA* transcription at least two-fold in vivo. BLM is a component of active RNA polymerase I transcription complexes. Disruption of transcription by the RNA polymerase I inhibitor actinomycin D re-distributes both RNA polymerase I components and BLM from the nucleolus into the nucleoplasm. In vitro, BLM facilitates unwinding of RNA:DNA hybrids (R loops) that have the potential to block ongoing *rDNA* transcription. This activity is enhanced by its association with TOP1, another integral component of the *rDNA* transcription machinery [36]. TOP1 is localized in the nucleolus and facilitates *rDNA* transcription by removing superhelical tension (relaxation) generated by ongoing transcription [37,38,39]. Our studies showed that BLM interacts with TOP1 via the C-terminus and enhances its relaxation activity by 3–4 fold in vitro [36]. These studies suggest collaborating functions of BLM and TOP1 in the nucleolus. They are also consistent with the inhibition of RNA polymerase I transcription in yeast deficient for the *BLM* ortholog *Sgs1* and *Srs2*, another 3′–5′ helicase, suggesting a conserved role for recQ-like helicases in nucleolar rRNA transcription [40].

In addition to RNA polymerase I components, BLM interacts with several other proteins that function in nucleolar metabolism, including nucleophosmin, DNA topoisomerase IIα (TOP2A), WRN and BRCA1 [35,41,42,43,44,45,46]. BLM also interacts with p53, a major stress sensor that controls the cellular response to nucleolar stress [10]. The functional implications of these interactions are unclear but suggest a nucleolar specific role for BLM in regulating multiple aspects of rDNA metabolism, including recombination, replication and transcription. Signals regulating nucleolar trafficking and/or targeting of BLM and their underlying mechanism(s) are unknown. The goal of this study is to delineate these signals within BLM with the long-term goal of understanding the basis of growth defects associated with BS and developing therapeutic interventions that use nucleolar BLM to overcome growth defects or prevent tumor growth. Our results map the TOP1-interaction region of BLM, identify specific amino acids that control its nucleolar trafficking and functionally characterize the role of these amino acids in BLM. Our results identify a putative phosphorylation mechanism that regulates nucleolar entry (and exit) of BLM.

## 2. Materials and Methods

### 2.1. Cell Lines

GM08505 immortalized BS fibroblasts were obtained from Coriell Cell Repository (Camden, NJ, USA). Cells were cultured in Minimum Essential Medium (Invitrogen, Carlsbad, CA, USA) supplemented with 10% fetal bovine serum (Hyclone, Logan, UT, USA) at 37 °C and 5% CO_2_.

### 2.2. Cloning

The *pYES-BLM* yeast expression vector was provided by Ian Hickson [47]. *BLM* deletion and site-specific mutants were generated by an overlapping polymerase chain reaction (PCR) with the *pYES-BLM* vector as a template using Phusion High-Fidelity DNA Polymerase (NEB, Ipswich, MA, USA). 3′ internal primers were used with the 5′ external primer; 5′ internal primers were used with the 3′ external primer. Corresponding products from both reactions were gel-purified (Qiagen, Hilden, Germany) and used as templates in an overlapping PCR with the 5′ external and 3′ external primers in a 1:1 ratio. Primers (5′–3′) used in *BLM* cloning were:

5′ external primer: CAGCTTTTGGCCTACTTTGGT
3′ external primer: TTCCTTTTCGGTTAGAGCGGA
3′ 1332-1349 deletion internal primer: CCACTGGAAGCAGTTCTGGTTTTACTTGCAAAGTAGTGG
5′ 1332-1349 deletion internal primer: CAAGTAAAACCAGAACTGCTTCCAGTGGTTCCAA
3′ S1342A/S1345A internal primer: CTCTTAGCCCTTTGGGCGGCTGG
5′ S1342A/S1345A internal primer: CCAGCCGCCCAAAGGGCTAAGAG
3′ S1342D/S1345D internal primer: CTCTTATCCCTTTGGTCGGCTGGC
5′ S1342D/S1345D internal primer: GCCAGCCGACCAAAGGGATAAGAG
3′ S1342A internal primer: CTCTTAGACCTTTGGGCGGCTGG
5′ S1342A internal primer: CCAGCCGCCCAAAGGTCTAAGAG
3′ S1345A internal primer: CTCTTAGCCCTTTGGGAGGCTGG
5′ S1345A internal primer: CCAGCCTCCCAAAGGGCTAAGAG
3′ S1342D internal primer: CTCTTAGACCTTTGGTCGGCTGGC
5′ S1342D internal primer: GCCAGCCGACCAAAGGTCTAAGAG
3′ S1345D internal primer: CTCTTATCCCTTTGGGAGGCTGGC
5′ S1345D internal primer: GCCAGCCTCCCAAAGGGATAAGAG

All restrictions enzymes were purchased from NEB. Gel-purified PCR products and *pYES-BLM* were digested with SalI and XbaI at 37 °C for 1.5 h per manufacturer’s instructions and gel-purified. T4 DNA ligase (NEB) was used in ligation reactions per manufacturer’s instructions. Ligation reactions were transformed into Dh5α-competent cells. Plasmids were purified with QIAprep Spin Miniprep kit (Qiagen) and E.Z.N.A. Endo-Free Plasmid Maxi Kit (Omega Bio-tek, Norcross, GA, USA). The full sequence of each *BLM* mutant was verified by the OSU Nucleic Acids Shared Resource. Inserts were transferred into the *pEGFP-BLM* vector, originally generated by cloning *BLM* cDNA into a mammalian expression vector containing an N-terminal GFP tag, *pEGFP-C1* (Clontech, Mountain View, CA, USA). The *pYES-BLM* mutants and *pEGFP-BLM* vector were digested with AflII and AsiSI at 37 °C for 1 h, and the remaining steps were carried out as previously described.

*TOP1* cDNA was purchased from Thermo Fisher Scientific (Waltham, MA, USA) as *pCR4-TOPO* and sub-cloned into the *pYES* yeast expression vector. A *Sac*I restriction site and His tag were added to the 5′ end of the cDNA and a *Sal*I site added to the 3′ end through PCR. Primers (5′–3′) used in TOP1 sub-cloning were:

5′ TOP1 primer: CGTAGAGCTCGGATCCCTAACCATGCACCACCACCACCACCACAGTGGGGACCACCTC
3′ TOP1 primer: TACGCTCGAGTCAAAACTCATAGTCTTCATCAGCC

Gel-purified PCR products were digested sequentially with SalI at 37 °C for 1 h and SacI at 37 °C for 1 h and purified with QIAquick PCR Purification Kit (Qiagen) in between digests. *pYES-BLM* vectors were digested with SacI and XhoI at 37 °C for 1 h. Remaining steps were carried out as previously described. The full sequence of *TOP1* cDNA was verified by the OSU Nucleic Acids Shared Resource.

### 2.3. In Vitro Transcription and Translation

In vitro transcription and translation (IVTT) reactions were performed with the TNT Rabbit Reticulocyte Lysate kit (Promega, Madison, WI, USA) according to manufacturer’s instructions with ^35^S-methionine. Peptides generated with the included luciferase control DNA were used as a negative control in subsequent immunoprecipitation reactions. DNA templates containing the indicated amino acids of BLM were generated by PCR using *pYES-BLM* as a template. The *Δ1332–1349* DNA template was generated by PCR using the *pYES-BLM-Δ1332*–*1349* plasmid as a template. The T7 promoter and a poly-A tail were added by PCR. PCR products were separated on agarose gels and purified with a QIAquick gel extraction kit (Qiagen). IVTT products were incubated with full-length recombinant TOP1 followed by α-TOP1 (A302-589A, Bethyl, Montgomery, TX, USA) or isotype control (rabbit IgG, P120-201, Bethyl) antibodies at 4 °C for a total of 3 h with rotation in buffer containing 10 mM Tris-HCl (pH 7.5), 150 mM NaCl, 1 mM EDTA, 10% glycerol, 1 mM DTT, 0.1% Tween-20, 0.1 mg/ml BSA and protease inhibitor (Sigma-Aldrich, St. Louis, MO, USA). Complexes were immunoprecipitated with Dynabeads Protein G (Invitrogen) at 4 °C for 2 h with rotation. Beads were washed 4 times for 5 min at 4 °C with rotation, and products were eluted with SDS-PAGE sample buffer at 95 °C for 5 min and separated by SDS-PAGE. Gels were dried, exposed to storage phosphor screens (GE, Fairfield, CT, USA), imaged with Typhon FLA 7000 (GE) and analyzed using ImageQuant software (GE). The C-terminus of BLM (amino acids 997–1417) was used as a positive control and luciferase as a negative control for each experimental replicate. Peptide fragment pulldowns were quantified, and non-specific pulldown by the IgG control antibody was subtracted. Each fragment was normalized to its input and expressed as % relative IP compared to the positive control (amino acids 997–1417). Experiments were repeated 3 to 9 times per peptide. Data were analyzed with a Student’s *t*-test (Graphpad Software, La Jolla, CA, USA).

### 2.4. Protein Purification

BLM was purified as previously described with the addition of a heparin-sepharose column [35,36,44]. Briefly, His-tagged *BLM* (*pYES-BLM-WT*, *pYES-BLM-Δ1332–1349*, *pYES-BLM*-*S1342A/S1345A* and *pYES-BLM-S1342D/S1345D*) and *TOP1* (*pYES-TOP1*) were expressed in *Jel1* *Saccharomyces cerevisiae*. Yeast cells were lysed with a French Press Cell Disrupter (Thermo Fisher Scientific), and cell debris was pelleted by ultracentrifugation at 65,000 g for 1 h at 4 °C. BLM protein was purified from the lysate by FPLC using Ni-NTA Superflow (Qiagen), Heparin-Sepharose 6 Fast Flow (Amersham Biosciences, Little Chalfont, UK) and Q-Sepharose (Sigma-Aldrich) columns with dialysis between each column in 50 mM Tris-HCl (pH 7.5), 200 mM NaCl, 1 mM EDTA, 10% glycerol, 1 mM DTT, protease inhibitor (Sigma-Aldrich) and 100 mM PMSF. The purified protein was dialyzed in 50 mM Tris-HCl (pH 7.5), 200 mM NaCl, 1 mM EDTA, 20% glycerol, 1 mM DTT, protease inhibitor (Sigma-Aldrich) and 100 mM PMSF, snap frozen in liquid N_2_ and stored at −80 °C. The *BLM* mutants eluted at similar salt concentrations as wild-type. TOP1 was purified similarly to BLM by FPLC using Ni-NTA Superflow (Qiagen) followed by Heparin-Sepharose 6 Fast Flow (Amersham Biosciences) columns with dialysis between columns. TOP1 eluted at 140–230 mM imidazole from Ni-NTA, and at 610–670 mM NaCl from the Heparin-Sepharose column. Purified TOP1 was dialyzed in 50 mM Tris-HCl (pH 7.5), 200 mM NaCl, 1 mM EDTA, 30% glycerol, 1 mM DTT, protease inhibitor (Sigma-Aldrich) and 100 mM PMSF, snap frozen in liquid N_2_ and stored at −80 °C. Single-band purity of the resulting proteins was verified by Coomassie blue staining of SDS-PAGE gels. Protein concentration was measured using Qubit Protein Assay Kit (Thermo Fisher Scientific).

### 2.5. Helicase Assays

DNA oligonucleotides were purchased from Operon (Huntsville, AL, USA), and RNA oligonucleotides were purchased from Invitrogen. Oligonucleotide sequences (5′ to 3′) are:

DNA_20_: CGCTAGCAATATTCTGCAGC
RNA_20_: CGCUAGCAAUAUUCUGCAGC
DNA_33_: GCTGCAGAATATTGCTAGCGGGAATTCGGCGCG

Oligonucleotides (DNA_20_ and RNA_20_) were end-labeled with ^32^P using polynucleotide kinase (NEB) according to manufacturer’s instructions and annealed with the DNA_33_ oligonucleotide by heating to 95 °C for 5 min followed by slow cooling to room temperature (RT), to generate the corresponding DNA:DNA or RNA:DNA substrates. Double-stranded substrate was separated on non-denaturing polyacrylamide gels and gel-purified. Helicase assays were performed with the indicated concentration of BLM protein and with the indicated substrate (DNA:DNA for protein activity determination or RNA:DNA for TOP1 stimulation experiments) as previously described [35,36,48]. Briefly, BLM protein was incubated with 2 fmol substrate, with and without a two-fold molar excess of TOP1, for 0.25, 0.5, 0.75, 1, 2, 3, 7 and 12 min at 37 °C in buffer containing 20 mM Tris-HCl (pH 7.5), 100 mM NaCl, 2 mM MgCl_2_, 2 mM ATP, 1 mM DTT and 0.1 mg/ml BSA. Reactions were stopped at the respective times and products separated on 12% non-denaturing polyacrylamide gels, dried, exposed to storage phosphor screens (GE), imaged with Typhon FLA 7000 (GE) and analyzed using ImageQuant software (GE). TOP1 alone did not unwind the RNA:DNA substrate (data not shown) [31]. Unwinding was quantified as the amount of single-stranded substrate relative to the total substrate per time-point after subtracting for single-stranded substrate in the minus BLM reaction. Substrate unwound (fmol) was plotted as a function of time and curves fitted to hyperbolic plots corresponding to the Michaelis-Menten equation with KaleidaGraph software (Synergy Software, Reading, PA, USA). Specific activities of the proteins, in units of fmol substrate unwound/min/nM protein, were calculated as the slope of the line of the initial unwinding rate plotted versus the protein concentration. Experiments were repeated 3 to 6 times for each protein concentration. Helicase assays using recombinant TOP2A (purchased from TopoGEN, Buena Vista, CO, USA) and BLM proteins were performed for 10 min at 37 °C in buffer containing 50 mM Tris-HCl (pH 8.0), 120 mM NaCl, 10 mM MgCl_2_, 1 mM ATP, 0.5 mM DTT and 50 μg/mL BSA, using radioactively labeled DNA:DNA substrate prepared by annealing the following oligonucleotides, as described [45]:

TTTTTTTTTTTTTTTTTAGGGTTAGGGCATGCACTAC
GTAGTGCATGCCCTAACCCTAATTTTTTTTTTTTTTT

Reactions were terminated in TOP2A stop buffer (1% Sarkosyl, 5% Glycerol, 10 mM EDTA); products were resolved on 10% non-denaturing polyacrylamide gels and quantified as described above.

### 2.6. Alignment and in Silico Phosphorylation Prediction

BLM protein sequences of different species were extracted from Ensembl and aligned using ClustalW 1.83 through the European Bioinformatics Institute. The output was re-formatted in MView, using default parameters. Identities were normalized by aligned length. Residues were colored by identity and property. BLM amino acids 1332 to 1349 were analyzed with the following programs: KinasePhos2.0 [49], NetPhorest [50], ScanSite3 [51], PPSP [52], Phosphonet [available online], and SABLE for Relative Solvent Accessibility (RSA) [54].

### 2.7. Immunofluorescence

GM08505 cells were plated on sterile coverslips and transfected with the indicated GFP-tagged BLM plasmids (pEGFP-BLM-wild-type, pEGFP-BLM-D795A, pEGFP-BLM-S1342A/S1345A, pEGFP-BLM-S1342D/S1345D, pEGFP-BLM-S1342A, pEGFP-BLM-S1345A, pEGFP-BLM-S1342D, pEGFP-BLM-S1345D and pEGFP-BLM-Δ1332–1349) with Lipofectamine2000 (Thermo Fisher Scientific) according to the manufacturer’s instructions. Cells were fixed with 4% paraformaldehyde for 15 min 24-h post-transfection, washed with Dulbecco’s phosphate-buffered saline (DPBS) 3 times for 5 min, permeabilized with 0.25% Triton-X-100 for 15 min, washed with DPBS 3 times 5 min and blocked with 10% normal goat serum (Sigma-Aldrich) (all steps were performed at RT). Anti-nucleophosmin (anti-B23, sc-47725, Santa Cruz, Dallas, TX, USA) was used at 1:1000, anti-PML (sc-966, Santa Cruz) was used at 1:500 and Alexa-Fluor (Invitrogen) fluorescent goat anti-mouse was used at 1:2000 in DPBS with 1% BSA (Sigma-Aldrich) and 0.1% Tween 20 (Sigma-Aldrich). Coverslips were mounted with VectaShield plus DAPI mounting medium (Vector Labs, Burlingame, CA, USA). Slides were imaged on a Zeiss AxioVert 200 M microscope and AxioCam MRm camera or an Olympus FV1000 filter confocal microscope. Nucleolar localization for 100 cells from 3 to 5 blinded experiments was expressed as percent nucleolar localization. PML body localization for 50 cells from 3 blinded experiments was expressed as percent PML body localization. Data were analyzed with a Student’s *t*-test (Graphpad Software, La Jolla, CA, USA).

## 3. Results

### 3.1. BLM Amino Acids 1332–1349 Are Required for Binding to TOP1

**Figure 1:**
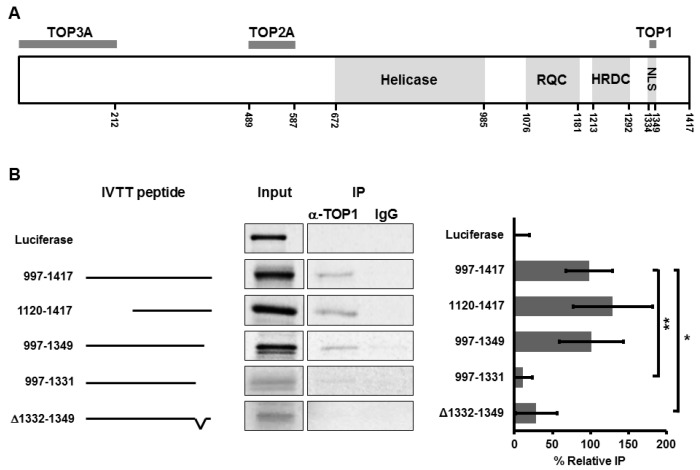
BLM amino acids 1332–1349 are required for binding to topoisomerase I. (**A**) Diagram of the full-length BLM protein (1417 amino acids) highlighting known functional domains and the interaction regions with topoisomerase I (TOP1), topoisomerase IIα (TOP2A) and topoisomerase IIIα (TOP3A). An additional TOP3A-interaction region consisting of amino acids 1266–1417 was reported in one study [56]; (**B**) ^35^S-methionine-labeled BLM peptide fragments of the indicated amino acids were generated using IVTT, incubated with purified TOP1 and immunoprecipitated using α-TOP1 or isotype control antibodies with Dynabeads Protein G. The C-terminus of BLM (amino acids 997–1417) was used as a positive control and luciferase as a negative control for each replicate. Pulldown of each peptide fragment was quantified and non-specific pulldown subtracted. Each fragment was normalized to its input and expressed as % relative IP compared to the positive control (amino acids 997–1417). Results from 3–9 experiments per peptide were compared to the positive control 997–1417 fragment using a Student’s *t*-test. A representative gel is shown for each. Error bars depict standard deviation. * *p* < 0.006, ** *p* < 0.001.

Previous studies showed that BLM and TOP1 collaborate in facilitating rRNA transcription [35,36]. The two proteins directly interact and reciprocally stimulate each’s enzymatic function. Most likely, this interaction promotes transcription, as in vitro TOP1 stimulates BLM helicase activity in unwinding RNA:DNA duplexes resembling the R loops formed during *rDNA* transcription, while BLM stimulates TOP1 activity in relaxation of helical tension generated in DNA during ongoing transcription [36]. To characterize these interactions, we identified the TOP1-interaction domain of BLM using co-immunoprecipitation of full-length recombinant TOP1 (Figure S1) with ^35^S-methionine-labelled BLM fragments generated by in vitro transcription and translation (IVTT). The BLM protein contains a number of domains (Figure 1A), including the helicase domain (amino acids 672–985), the recQ C-terminal domain (RQC, amino acids 1076–1181), that functions in DNA recognition and binding, the helicase and RNase D C-terminal domain (HRDC, amino acids 1213–1292), that controls substrate recognition, and a nuclear localization sequence (NLS, amino acids 1334–1349) [55]. Our earlier work showed that the TOP1-interaction domain in BLM resides in the C-terminus (amino acids 997–1417) [36]. As the efficiency of IVTT of full-length BLM is poor due to its size, the C-terminal fragment (997–1417) was used as a positive control for co-immunoprecipitation with full-length, purified TOP1 protein. Luciferase was selected as a negative control. Sequential deletions were made within the 997–1417 fragment of BLM, and each was tested for its ability to interact with TOP1 (Figure 1B). Results show that deletion of amino acids 997–1119 at the N-terminus of this fragment or amino acids 1350–1417 at the extreme C-terminus of the fragment does not alter the ability of BLM to interact with TOP1 (Figure 1B, compare 997–1417 with 1120–1417 and 997–1349). Deletion of amino acids 1332–1417 reduced interaction with TOP1 to negligible levels (Figure 1B, compare 997–1349 with 997–1331), indicating that TOP1 interaction requires amino acids 1332–1349. Amino acids 1332–1349 were then deleted from the C-terminal fragment 997–1417 (yielding the fragment Δ1332–1349) to confirm that amino acids 1332–1349 are required for TOP1 interaction (Figure 1B, compare 997–1417 with Δ1332–1349). Since this region also includes the BLM NLS, our results suggest an additional function for the region in TOP1 interaction. The TOP1-interaction region is distinct from the TOP2A [44] and DNA topoisomerase III alpha- (TOP3A) [56,57,58] interaction regions (Figure 1A).

### 3.2. Deletion of Amino Acids 1332–1349 in BLM Abolishes TOP1-Mediated Unwinding of RNA:DNA Substrate

**Figure 2:**
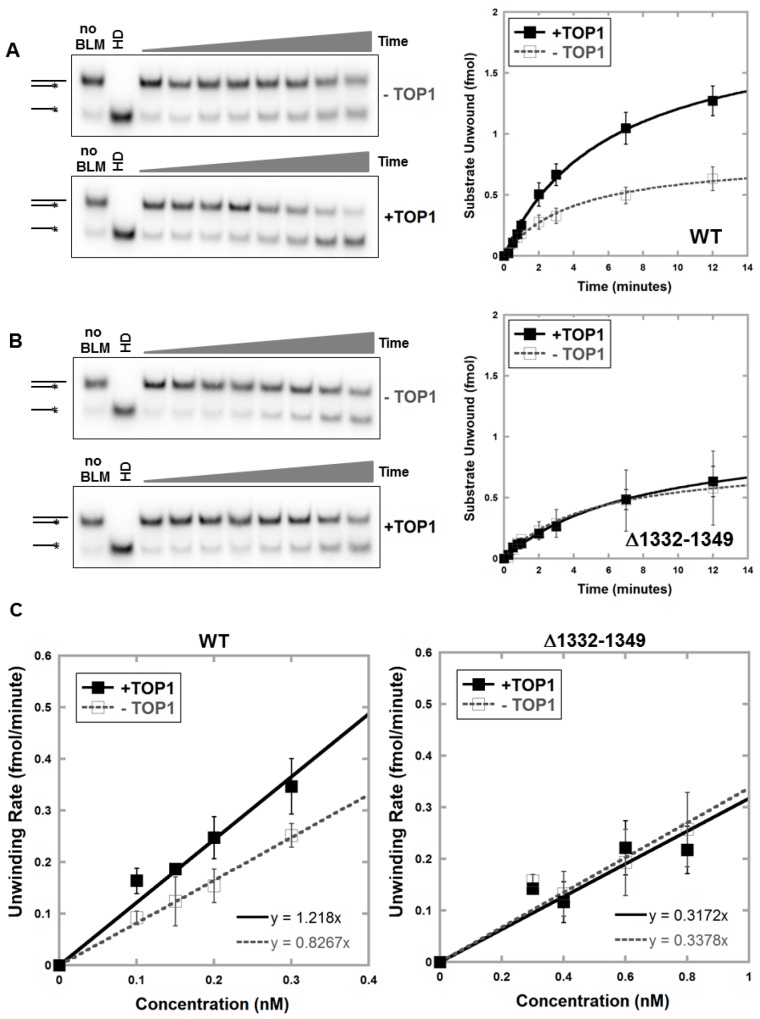
Unwinding of RNA:DNA substrate by BLM-Δ1332–1349 is not stimulated by TOP1. Kinetics of unwinding of RNA:DNA substrate was assessed using a range of BLM protein concentrations with and without TOP1. Representative gels and kinetics of unwinding for one representative protein concentration, 0.2 nM BLM-WT (**A**) or 0.3 nM BLM-Δ1332–1349 (**B**) are shown. BLM protein with two-fold molar excess TOP1 or equivalent volume of reaction buffer was incubated with 2 fmol RNA:DNA substrate for 0.25, 0.5, 0.75, 1, 2, 3, 7 and 12 min at 37 °C, and double- and single-stranded products were separated on non-denaturing acrylamide gels. Heat denatured (HD) substrate was generated by heating at 95 °C for 5 min. Unwinding was quantified as the amount of single-stranded substrate generated compared to the total substrate per time-point. Experiments were repeated 3 to 6 times for each protein concentration, and curves showing amount of substrate unwound (fmol) as a function of time were fitted to hyperbolic plots corresponding to the Michaelis-Menten equation. Reactions with TOP1 alone were performed with each experiment to verify the lack of unwinding ability of TOP1. Error bars depict standard deviation. (**C**) Specific activities of BLM proteins with and without TOP1 were calculated by measuring initial unwinding rates for 4 protein concentrations (as in panels A and B; Figures S3 and S4) and graphed as a function of protein concentration; specific activity was calculated from the slope of the line (fmol substrate unwound/min/nM protein). Error bars depict standard deviation.

Recombinant BLM proteins were generated and their specific activity evaluated in vitro to confirm that BLM amino acids 1332–1349 are required for functional interaction with TOP1 (Figure 2). BLM wild-type (WT), BLM-Δ1332–1349 and TOP1 were expressed using yeast and purified (Figure S1). Helicase activity of purified BLM proteins was measured using ^32^P-labeled double-stranded DNA (dsDNA) substrate with a 13-base 3′ single-strand overhang. Initial unwinding rates for four protein concentrations were graphed to calculate the specific activities of the proteins, expressed in fmol substrate unwound/min/nM protein. BLM-Δ1332–1349 unwound dsDNA with a lower specific activity than BLM-WT (Figure S2, 1.5 vs. 2.9). Our previous studies demonstrated that TOP1 stimulates BLM unwinding of R loop-like GC-rich RNA:DNA substrates in vitro [35,36]. To determine if amino acids 1332–1349 are required for this stimulation, we measured unwinding activity of BLM-WT and BLM-Δ1332–1349 on RNA:DNA substrates with and without a two-fold molar excess of TOP1. The effect of TOP1 was measured at four different BLM protein concentrations based on the activity of each protein. Consistent with previous studies, BLM-WT was stimulated 1.5-fold with TOP1 (Figure 2A,C and Figure S3) [36]. However, BLM-Δ1332–1349 was not stimulated by TOP1 at any protein concentration (Figure 2B,C and Figure S4). To verify that the loss of stimulation of BLM-Δ1332–1349 is specific to TOP1, we tested the ability of TOP2A to stimulate BLM helicase activity. We previously demonstrated that TOP2A stimulates BLM unwinding of dsDNA and interacts with the N-terminus of BLM (amino acids 489–587) [44] BLM-Δ1332–1349 unwinding of dsDNA substrate was stimulated approximately 3-fold by TOP2A, similarly to BLM-WT (Figure S5). Our results are consistent with a requirement for amino acids 1332–1349 to facilitate a functional interaction of BLM and TOP1 in unwinding RNA:DNA duplexes.

### 3.3. Amino Acids in the Region 1332–1349 Are Critical for Nucleolar Targeting of BLM

BLM normally localizes within PML nuclear bodies and the nucleolus [33]. In order to assess the cellular consequences of deleting amino acids 1332–1349, GFP-tagged BLM constructs were transiently expressed in a BS fibroblast cell line, GM08505. Nucleolar localization was monitored using BLM co-localization with the nucleolar protein nucleophosmin (NPM). Studies with BLM-Δ1332–1349 revealed that it localizes primarily outside the nucleus (Figure S6). The TOP1-interaction region (1332–1349) also includes the arginine- and lysine-rich bipartite NLS, most likely preventing BLM-Δ1332–1349 from entering the nucleus. These data are consistent with previous analyses of the NLS [59,60].

**Figure 3:**
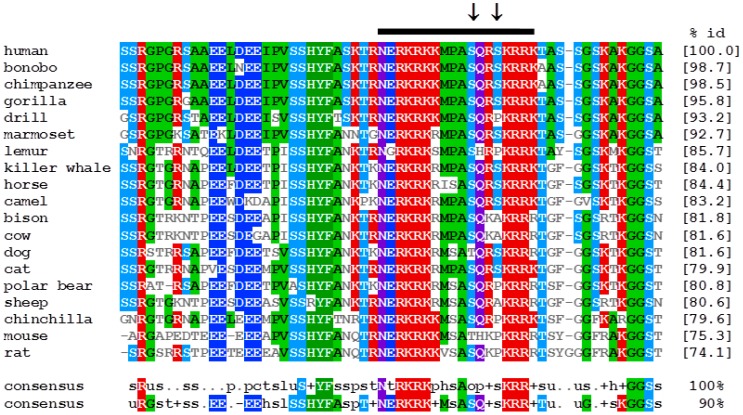
The TOP1-interaction domain of BLM is highly conserved in mammals. BLM protein sequences were extracted from Ensembl and aligned using ClustalW 1.83 at the European Bioinformatics Institute. The output was re-formatted in MView, using default parameters. Identities are normalized by aligned length. Residues are colored by identity and property. The TOP1-interaction region is indicated with a black bar and corresponds to human BLM residues 1332–1349. Serines 1342 and 1345 are highlighted with arrows. The nuclear localization sequence (NLS) region of BLM is contained within this region (1334–1349). The number in brackets at the end of each entry indicates percentage identity of the full-length protein sequence with respect to the human BLM protein. Consensus sequences for 90% and 100% identity are shown below. Accession numbers for the sequences are as follows: human [*Homo sapiens*; NP_000048.1]; bonobo [*Pan paniscus*; XP_003807768.1]; chimpanzee [*Pan troglodytes*; XP_510594.2]; lowland gorilla [*Gorilla gorilla gorilla*; XP_004056835.1]; drill [*Mandrillus leucophaeus*; XP_011833777.1]; marmoset [*Callithrix jacchus*; XP_002749167.1]; grey mouse lemur [*Microcebus murinus*; XP_012618727.1]; killer whale [*Orcinus orca*; XP_004278332.1]; horse [*Equus caballus*; XP_001502766.1]; Bactrian camel [*Camelus ferus*; XP_006192661.1]; bison [*Bison bison bison*; XP_010839366.1]; cow [*Bos Taurus*; XP_613809.3]; domestic dog [*Canis lupus familiaris*; XP_003434427.1]; domestic cat [*Felis catus*; XP_011281067.1]; polar bear [*Ursus maritimus*; XP_008686016.1]; sheep [*Ovis aries*; XP_004017810.1]; chinchilla [*Chinchilla lanigera*; XP_005381562.1]; mouse [*Mus musculus*; NP_031576.4]; rat [*Rattus norvegicus*; XP_003753349.2].

Therefore, a multiple alignment of the NLS and its flanking regions was compiled to assess conservation of the amino acids within the TOP1-interaction region (Figure 3). Analyses demonstrate that the arginine- and lysine-rich bipartite motifs are completely conserved among 19 selected mammalian species. Although there is more variability in the composition of the other residues, all of the included species contain one (drill, mouse, cow) to three (horse) serines in this region. The dog and mouse sequences are notable in that each possesses a single threonine residue that aligns with an otherwise completely conserved serine (human BLM S1342). It is known that physiological functions of BLM are regulated by phosphorylation by several serine/threonine kinases [61]. The BLM 1332–1349 region was thus analyzed with multiple programs in silico to identify potential phosphorylation sites. NetPhos2.0 identified S1342 and S1345 as putative phosphorylation sites with high probability (score of 0.98); accessibility of this region to solvent was confirmed using SABLE. Multiple programs predict that PKC or the DNA damage-sensitive kinases ATM and ATR are highly likely to phosphorylate these residues. Given that this region is highly conserved among mammalian BLM proteins, these analyses suggest that additional functions of the NLS in facilitating protein-protein interactions are likely to be conserved (Figure 3). In the aligned region shown, the NLS sequences contain 1–3 serine residues that are putative targets for post-translational modification by cellular kinases, including ATM, PKA and AKT1, suggesting that these sites could control *rDNA* stability as well as cellular growth.

**Figure 4:**
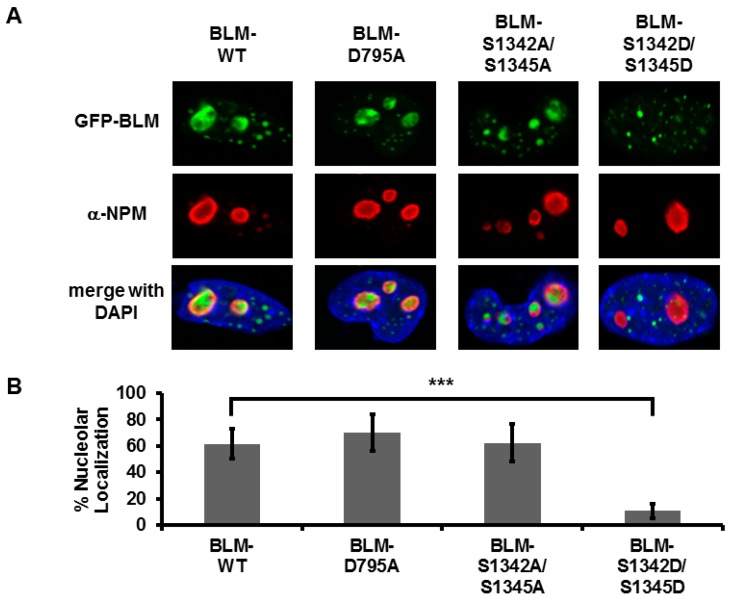
S1342 and S1345 determine nucleolar localization of BLM. (**A**) Cellular localization of BLM phospho-dead (BLM-S1342A/S1345A) and phospho-mimetic (BLM-S1342D/S1345D) mutants. GM08505 *BLM*^−/−^ cells were plated on sterile coverslips and transfected with the indicated GFP-tagged *BLM* plasmids, *BLM-WT*, *BLM-D795A* (helicase dead), *BLM-S1342A/S1345A* and *BLM-S1342D/S1345D*, using Lipofectamine 2000. Cells were fixed with 4% paraformaldehyde 24-h post-transfection, permeabilized with 0.25% Triton-X-100 and blocked with 10% normal goat serum. Nucleoli were stained with anti-nucleophosmin (α-NPM) and Alexa-Fluor fluorescent secondary antibodies and coverslips were mounted with VectaShield plus DAPI mounting medium. (**B**) Quantification of nucleolar localization. Nucleolar localization for 100 cells from 4 to 5 blinded experiments was expressed as percent nucleolar localization. Error bars depict standard deviation. Results were compared to *BLM-WT* using a Student’s *t*-test (*** *p* < 0.0001).

**Figure 5:**
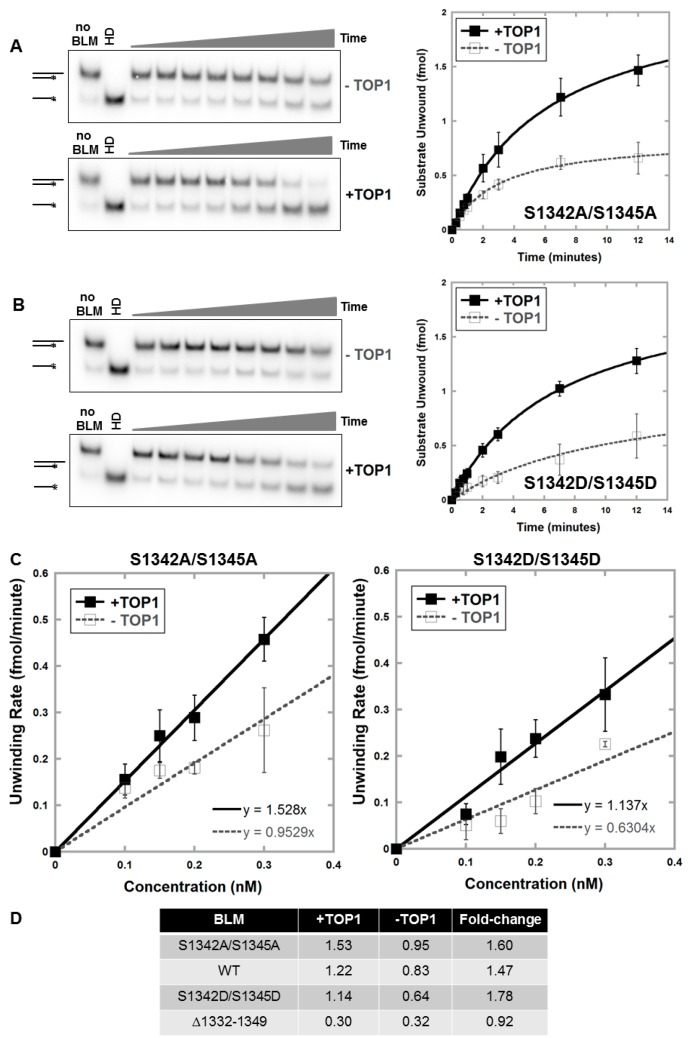
Unwinding of RNA:DNA substrate by BLM-S1342A/S1345A or BLM-S1342D/S1345D is equally stimulated by TOP1. Kinetics of unwinding of RNA:DNA substrate were assessed using a range of BLM protein concentrations with and without TOP1. Representative gels and kinetics of unwinding for one representative protein concentration, 0.2 nM, by BLM-S1342A/S1345A (**A**) or BLM-S1342D/S1345D (**B**) are shown. BLM protein with two-fold molar excess of TOP1 or equivalent volume of reaction buffer was incubated with 2 fmol RNA:DNA substrate for 0.25, 0.5, 0.75, 1, 2, 3, 7 and 12 min at 37 °C; double- and single-stranded products were separated on non-denaturing acrylamide gels. Heat denatured (HD) substrate was generated by heating at 95 °C for 5 min. Unwinding was quantified as the amount of single-stranded substrate compared to the total substrate per time-point. Experiments were repeated 3 to 6 times for each protein concentration, and curves showing amount of substrate unwound (fmol) as a function of time were fitted to hyperbolic plots corresponding to the Michaelis-Menten equation. BLM-WT was used as a positive control for the ability of TOP1 to stimulate BLM (Figure 2). Reactions with TOP1 alone were performed with each experiment to verify the lack of unwinding ability of TOP1. Error bars depict standard deviation. (**C**) Specific activities of BLM proteins with and without TOP1 were calculated by measuring initial unwinding rates for 4 protein concentrations (as in panels A and B; Figures S9 and S10) and graphed as a function of protein concentration; specific activity was calculated from the slope of the line (fmol substrate unwound/min/nM protein) (Figures S7 and S8). Error bars depict standard deviation. (**D**) Table showing the fold-change of RNA:DNA unwinding activity of each BLM protein with TOP1. Specific activities shown (fmol/min/nM) were obtained as in panel C and Figure 2C.

S1342 and/or S1345 were mutated to alanine (phospho-dead) or aspartic acid (phospho-mimic) and tested for their ability to alter nucleolar localization using GFP-tagged constructs (Figure 4) or to interact with TOP1 using purified proteins (Figure 5). The helicase-dead mutant of BLM (BLM-D795A) was used as a control for mutation in a domain separate from TOP1 interaction. Co-localized foci were quantitated and represented as percent nucleolar localization. BLM-WT and BLM-D795A localized similarly to the nucleolus with about 60% of cells positive for nucleolar BLM. Alanine mutagenesis of the two serines (BLM-S1342A/S1345A) does not significantly alter nucleolar localization of BLM compared to BLM-WT or the helicase-dead mutant (Figure 4). In contrast, aspartic acid mutagenesis of the serines (BLM-S1342D/S1345D) significantly reduces nucleolar targeting (*p* < 0.0001, Figure 4). Mutagenesis of S1342 and S1345 to aspartic acid or alanine does not affect BLM localization to PML bodies (Figure S7). Single mutants display similar patterns of nucleolar localization to corresponding double mutants (*p* > 0.05; Figure S8). Our results suggest that S1342 and S1345 are critical for nucleolar trafficking, but do not alter localization to the nucleus or PML bodies, and implicate the NLS region in TOP1 interaction and nucleolar trafficking.

### 3.4. Alanine or Aspartic acid Mutagenesis of S1342 and S1345 Does Not Alter the Functional Interaction of BLM and TOP1

Recombinant mutant proteins BLM-S1342A/S1345A and BLM-S1342D/S1345D were purified and characterized (Figures S1 and S2) and unwinding activities measured to assess whether S1342 and S1345 mutation alters TOP1 stimulation of BLM activity with RNA:DNA duplexes (Figure 5, Figures S9 and S10). Results show that neither alanine nor aspartic acid mutations alter TOP1 stimulation of BLM compared to wild-type BLM (Figure 5) and suggest that these serines do not affect the functional interaction of TOP1 and BLM but modify BLM nucleolar entry or exit.

## 4. Discussion and Conclusions

Unlike nuclear localization sequences (NLS), nucleolar localization sequences are not clearly defined. Both are composed of a high proportion of basic amino acids and may either be distinct or overlap within any one protein [62]. Known mechanisms that localize proteins to the nucleolus include protein-protein interactions with a nucleolar protein, protein-*rDNA* or protein-rRNA interactions and protein-small nucleolar RNA interactions [63,64]. BLM binds *rDNA* via its C-terminus; regions overlapping the RQC (amino acids 1118–1164) and HRDC domains (amino acids 1166–1331) mediate these interactions [31,32,33]. The C-terminus (amino acids 997–1417) likewise mediates the interaction of BLM with TOP1, also enriched in the nucleolus [35,36]. The data presented here further refine the TOP1-interaction domain of BLM. We show that the region necessary for physical and functional interaction with TOP1 resides within residues 1332–1349 (Figure 1 and Figure 2). The TOP1-interaction region of BLM is distinct from the TOP2A- and TOP3A-interaction regions of BLM, as well as the residues directing BLM localization to PML bodies, primarily within the N-terminus [33,43,44,56,57,58,65]. Consequently, deletion of the TOP1-interaction region of BLM does not impair the functional interaction with TOP2A (Figure S5). One study [56] suggested an additional TOP3A-interaction region in the C-terminus of BLM (amino acids 1266–1417) that overlaps with the TOP1-interaction region; the effect of the deletion of 1332–1349 or mutations within this region (S1342 and S1345) on the TOP3A interaction is unclear. The TOP1-interaction region includes the NLS (1334–1349), a lysine- and arginine-rich basic region that is highly conserved among mammalian BLM homologues (Figure 3). We also demonstrate that S1342 and S1345 are critical for BLM nucleolar trafficking (Figure 4). While mutation of the serines to alanine does not change BLM function or localization, their mutation to phospho-mimic, aspartic acids, results in a dramatic reduction in nucleolar localization but not in nuclear localization, PML body localization or functional interaction with TOP1 (Figure 4 and Figure 5, Figure S7). Given that both the *rDNA* binding domains of BLM are intact in these mutants, our results argue that the effect of the phospho-mimetic modification in limiting nucleolar localization is dominant over the effects of any *rDNA* transactions or TOP1 interactions that might direct BLM to the nucleolus. Unimpaired localization of these mutants to PML bodies suggests that they would retain BLM functions in global DNA repair. The ability of the BLM NLS to direct TOP1 functional interactions as well as modulate nucleolar trafficking adds to the known functions of the BLM NLS.

Several studies have identified distinct sites within BLM that are modified post-translationally by phosphorylation, ubiquitination and sumoylation in response to cellular stress [61]. These modifications alter its physiological functions, its associations with numerous protein partners, localization to damaged sites upon stress or to PML bodies, chromatin localization, mitotic function or its stability [66,67,68,69,70,71,72,73,74,75,76,77,78,79,80,81,82,83,84]. Due to their highly repetitive nature and GC-rich regions, *rDNA* loci are prone to the formation of aberrant structures and damage that pose blocks to ongoing replication and transcription. BLM is an integral part of DNA damage repair pathways that promote resolution of such aberrant structures. In its absence or inappropriate localization, these transcription/replication blocks could elicit a DNA damage response that without BLM would increase recombination between *rDNA* repeats.

Accumulating evidence suggests a sub-nuclear spatial separation of the different aspects of *rDNA* metabolism—transcription, replication and recombination—to ensure stability of the *rDNA* loci. Early replicating *rDNA* loci (transcriptionally active) localize at the nucleolar periphery or outside the nucleolus during early S-phase and are subsequently internalized in the nucleolus [85]. Replication of transcriptionally silent *rDNA* loci occurs during mid- to late-S phase inside the nucleolus. Likewise, during recombinational-repair of double-strand breaks, there is a transient translocation of *rDNA* loci from the nucleolus to the nucleoplasm accompanied by a coordinated localization of recombination proteins to the nucleoplasm [86,87,88,89]. In contrast, *rDNA* transcription by RNA polymerase I is localized solely within the nucleolus. The dynamics of *rDNA* loci during the replication/transcription processes may preserve *rDNA* loci stability by preventing collisions of the transcriptional and replication machinery [85,88]. Serine modifications in the BLM NLS, potentially through phosphorylation, and the resulting change in BLM nucleolar trafficking presents an efficient mechanism for facilitating maintenance of *rDNA* loci by BLM. Likewise, restricting nucleolar entry of BLM may facilitate slower rates of *rDNA* transcription and the ensuing ribosome biogenesis, perhaps favorable under conditions of starvation or stress [6,9,13,35,36]. Several of the kinases predicted to phosphorylate BLM S1342 and S1345 and its orthologues (PKC, PKA and AKT) are sensitive to growth factors and regulate cell growth pathways. Our results suggest further study of the specific kinase-phosphatase pairs that may regulate BLM nucleolar trafficking. Such studies may also identify therapeutic strategies to modify BLM localization to control cell growth while preserving extra-nucleolar functions of BLM.

## Supplementary Materials

The following are available online at www.mdpi.com/2073-4425/7/9/69/s1.

Figure S1: Purification of recombinant BLM and TOP1 proteins. His-tagged BLM and TOP1 proteins were expressed in yeast and purified to homogeneity as described previously [36]. Protein purity is demonstrated by a single band using 8% SDS-PAGE and Coomassie Brilliant Blue staining.

Figure S2: Specific activity of purified BLM proteins. Kinetics of unwinding of DNA:DNA substrate were assessed using a range of BLM protein concentrations. Representative gels (**A**) and unwinding kinetics (**B**) for one representative protein concentration (0.3 nM) are shown for BLM-WT, BLM-Δ1332–1349, BLM-S1342A/S1345A and BLM-S1342D/S1345D. BLM protein was incubated with 2 fmol DNA:DNA substrate for 0.25, 0.5, 0.75, 1, 2, 3, 7 and 12 min at 37 °C; double- and single-stranded products were separated on non-denaturing acrylamide gels. Heat denatured (HD) substrate was generated by heating at 95 °C for 5 min. Amount of substrate unwound (fmol) was plotted as a function of time (minutes) and curves fitted to hyperbolic plots corresponding to the Michaelis-Menten equation. Experiments were repeated 3 to 6 times for each protein concentration. Error bars depict standard deviation. (**C**) Specific activities of BLM-WT, BLM-Δ 1332–1349, BLM-S1342A/S1345A and BLM-S1342D/S1345D were calculated by measuring initial unwinding rates for 4 protein concentrations and graphed as a function of protein concentration; specific activity was calculated from the slope of the line (fmol substrate unwound/min/nM protein). Error bars depict standard deviation. (**D**) Table showing summary of DNA:DNA unwinding activity of each BLM protein.

Figure S3: Unwinding of RNA:DNA substrate by BLM-WT is stimulated by TOP1. Changes in BLM-WT activity with a RNA:DNA substrate by TOP1 was assessed by quantifying the unwinding as a function of time using a range of BLM protein concentrations with and without TOP1, as in Figure 2A. Curves were fitted to hyperbolic plots corresponding to the Michaelis-Menten equation.

Figure S4: Unwinding of RNA:DNA substrate by BLM-Δ1332–1349 is not stimulated by TOP1. Changes in BLM-Δ1332–1349 activity with a RNA:DNA substrate by TOP1 was assessed by quantifying the unwinding as a function of time using a range of BLM protein concentrations with and without TOP1, as in Figure 2B. Curves were fitted to hyperbolic plots corresponding to the Michaelis-Menten equation.

Figure S5: Unwinding of DNA:DNA substrate by BLM-Δ1332–1349 is stimulated by TOP2A. Unwinding of DNA:DNA substrate was assessed using protein concentrations yielding similar unwinding activities (0.08 nM BLM-WT or 0.13 nM BLM-Δ1332–1349), with 7 nM TOP2A. Reactions were stopped after 10 min, and double- and single-stranded products were separated on non-denaturing acrylamide gels. Unwinding was quantified as the amount of single-stranded substrate generated compared to the total substrate, and results represented as fold-change relative to minus TOP2A. Experiments were repeated 4 times. BLM-WT and BLM-Δ1332–1349 were similarly stimulated by TOP2A (ns, not significant; *p* > 0.05). Error bars depict standard deviation. Statistical comparisons were made using a Student’s *t*-test.

Figure S6: BLM-Δ1332–1349 does not localize to the nucleus. GM08505 *BLM*^−/−^ cells were transfected with the indicated GFP-tagged *BLM* plasmids, *BLM-WT* and *BLM-**Δ1332–1349*, using Lipofectamine 2000. Cells were fixed with 4% paraformaldehyde 24-h post-transfection, and coverslips mounted with VectaShield plus DAPI-mounting medium. The figure shows that while BLM-WT localizes to the nucleus, BLM-Δ1332–1349 localizes primarily outside the nucleus with an insignificant number of foci within the nucleus.

Figure S7: S1342 and S1345 do not affect PML body localization of BLM. (**A**) Cellular localization of BLM phospho-dead (BLM-S1342A/S1345A) and phospho-mimetic (BLM-S1342D/S1345D) mutants. GM08505 *BLM*^−/−^ cells were plated on sterile coverslips and transfected with the indicated GFP-tagged *BLM* plasmids, *BLM-WT*, *BLM-S1342A/S1345A* and *BLM-S1342D/S1345D*, using Lipofectamine 2000. Cells were fixed with 4% paraformaldehyde 24-h post-transfection, permeabilized with 0.25% Triton-X-100 and blocked with 10% normal goat serum. PML bodies were stained with α-PML and Alexa-Fluor fluorescent secondary antibodies, and coverslips were mounted with VectaShield plus DAPI mounting medium. (**B**) Quantification of PML body localization. Co-localization of BLM and PML for 50 cells from 3 blinded experiments was expressed as percent PML body localization. All proteins had similar patterns of localization (ns, not significant; *p* > 0.05). Error bars depict standard deviation. Statistical comparisons were made using a Student’s *t*-test.

Figure S8: Nucleolar localization of BLM is affected by phospho-mimetic mutagenesis of BLM-S1342 and/or BLM-S1345. GM08505 *BLM*^−/−^ cells were transfected with the indicated GFP-tagged *BLM* plasmids, *BLM-WT*, *BLM-D795A* (helicase dead), *BLM-S1342A/S1345A*, *BLM-S1342A*, *BLM-S1345A*, *BLM-S1342D/S1345D*, *BLM-S1342D*, *BLM-S1345D* and empty vector using Lipofectamine 2000. Nucleolar localization of BLM mutants for 100 cells from 3 to 5 blinded experiments was expressed as % nucleolar localization, as in Figure 4. BLM proteins with mutated S1342D, S1345D and S1342D/S1345D amino acids demonstrated significant differences in nucleolar localization compared to BLM WT, *p* < 0.001, *p* < 0.001 and *p* < 0.0001, respectively. However, single BLM mutants of either alanine or aspartate presented similar patterns of nucleolar localization to their cognate double mutants (ns, not significant; *p* > 0.05). Error bars depict standard deviation. Statistical comparisons were made using a using a Student’s *t*-test.

Figure S9: Unwinding of RNA:DNA substrate by BLM-S1342A/S1345A is stimulated by TOP1. Changes in BLM-S1342A/S1345A activity with a RNA:DNA substrate by TOP1 was assessed by quantifying the unwinding as a function of time using a range of BLM protein concentrations with and without TOP1, as in Figure 5A. Curves were fitted to hyperbolic plots corresponding to the Michaelis-Menten equation.

Figure S10: Unwinding of RNA:DNA substrate by BLM-S1342D/S1345D is stimulated by TOP1. Changes in BLM-S1342D/S1345D activity with a RNA:DNA substrate by TOP1 were assessed by quantifying the unwinding as a function of time using a range of BLM protein concentrations with and without TOP1, as in Figure 5B. Curves were fitted to hyperbolic plots corresponding to the Michaelis-Menten equation.
