# Supplementary material for: Regulation of BLM Nucleolar Localization
Source: Genes (Basel). 2016 Sep 21;7(9):69. doi: 10.3390/genes7090069 (PMC5042399; doi:10.3390/genes7090069)
Supplement: Supplementary file 1 [file genes-07-00069-s001.docx]

Supplementary Materials: Regulation of BLM Nucleolar Localization

Larissa Tangeman, Michael A. Mcilhatton, Patrick Grierson, Joanna Groden and Samir Acharya


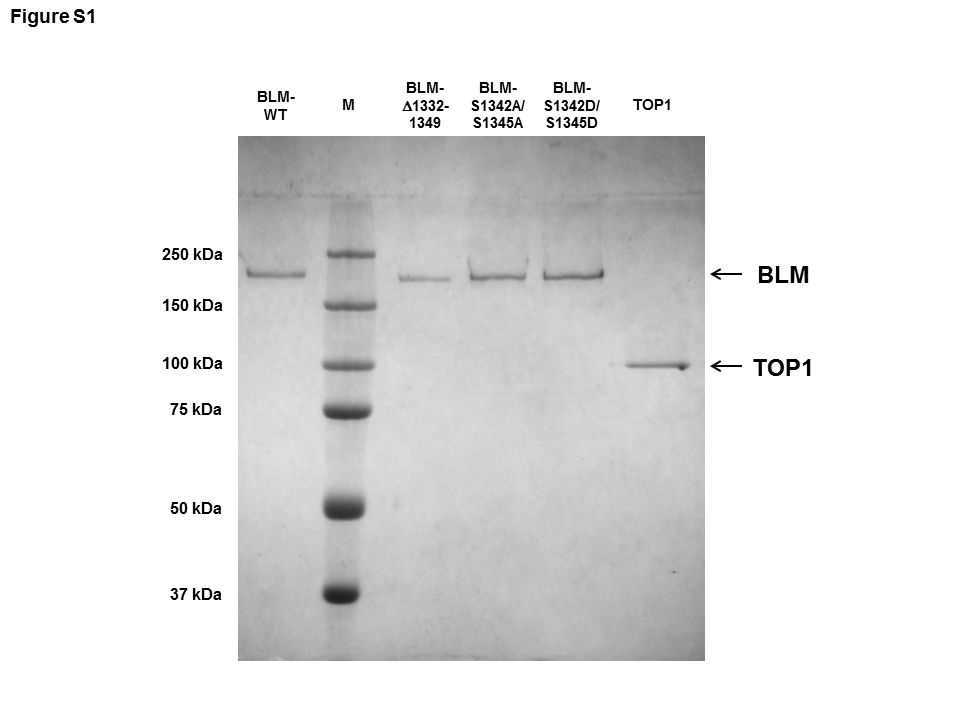


**Figure S1.** Purification of recombinant BLM and TOP1 proteins. His-tagged BLM and TOP1 proteins were expressed in yeast and purified to homogeneity as described previously [36]. Protein purity is demonstrated by a single band using 8% SDS-PAGE and Coomassie Brilliant Blue staining.


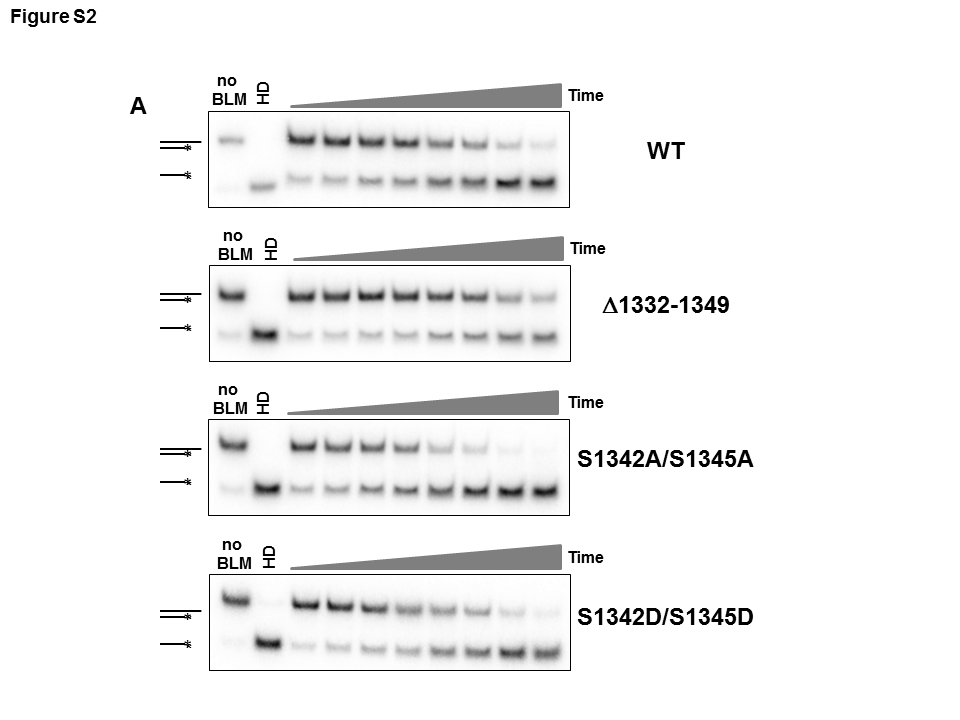


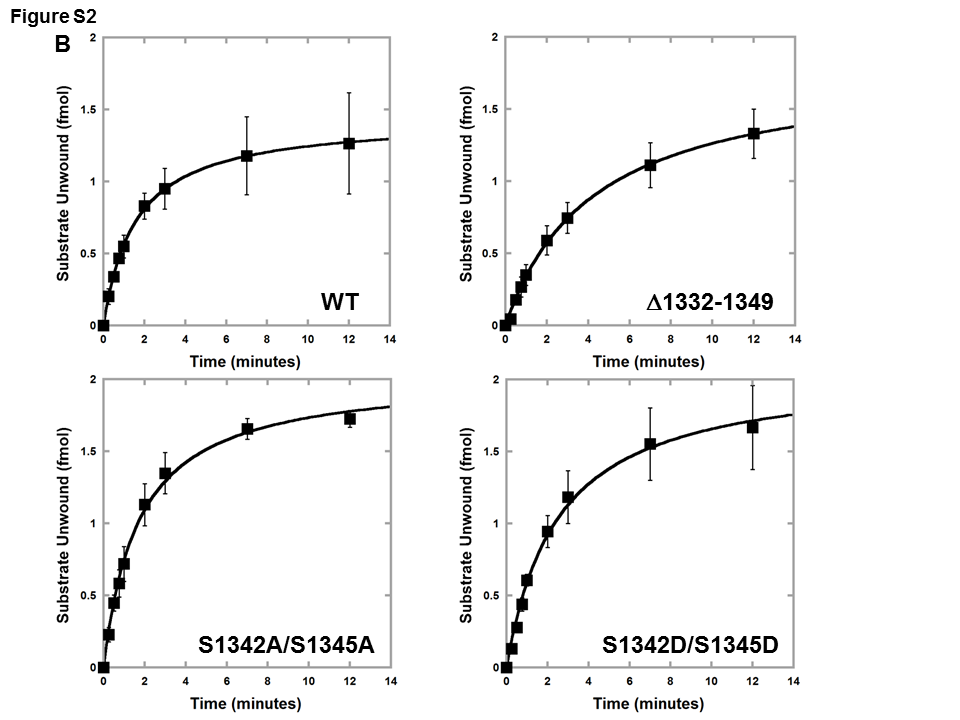


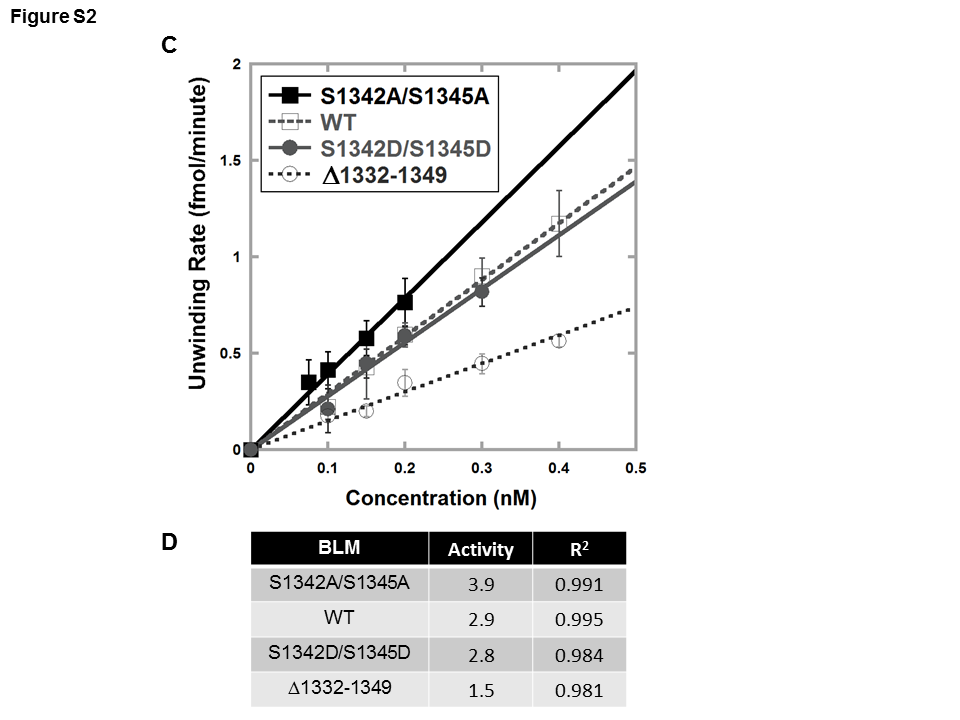


**Figure S2.** Specific activity of purified BLM proteins. Kinetics of unwinding of DNA:DNA substrate were assessed using a range of BLM protein concentrations. Representative gels (**A**) and unwinding kinetics (**B**) for one representative protein concentration (0.3 nM) are shown for BLM-WT, BLM-Δ1332-1349, BLM-S1342A/S1345A and BLM-S1342D/S1345D. BLM protein was incubated with 2 fmol DNA:DNA substrate for 0.25, 0.5, 0.75, 1, 2, 3, 7 and 12 minutes at 37 °C; double- and single-stranded products were separated on non-denaturing acrylamide gels. Heat denatured (HD) substrate was generated by heating at 95 °C for 5 min. Amount of substrate unwound (fmol) was plotted as a function of time (minutes) and curves fitted to hyperbolic plots corresponding to the Michaelis-Menten equation. Experiments were repeated 3 to 6 times for each protein concentration. Error bars depict standard deviation. (**C**) Specific activities of BLM-WT, BLM- Δ 1332-1349, BLM-S1342A/S1345A and BLM-S1342D/S1345D were calculated by measuring initial unwinding rates for 4 protein concentrations and graphed as a function of protein concentration; specific activity was calculated from the slope of the line (fmol substrate unwound/min/nM protein). Error bars depict standard deviation. (**D**) Table showing summary of DNA:DNA unwinding activity of each BLM protein.


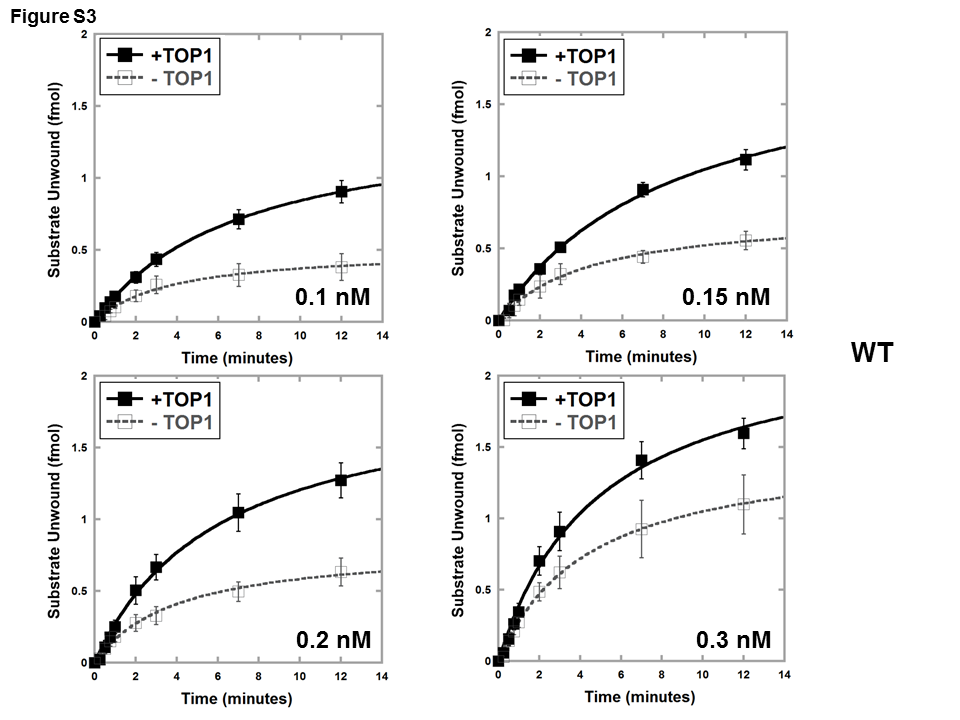


**Figure S3.** Unwinding of RNA:DNA substrate by BLM-WT is stimulated by TOP1. Changes in BLM-WT activity with a RNA:DNA substrate by TOP1 was assessed by quantifying the unwinding as a function of time using a range of BLM protein concentrations with and without TOP1, as in Figure 2A. Curves were fitted to hyperbolic plots corresponding to the Michaelis-Menten equation.


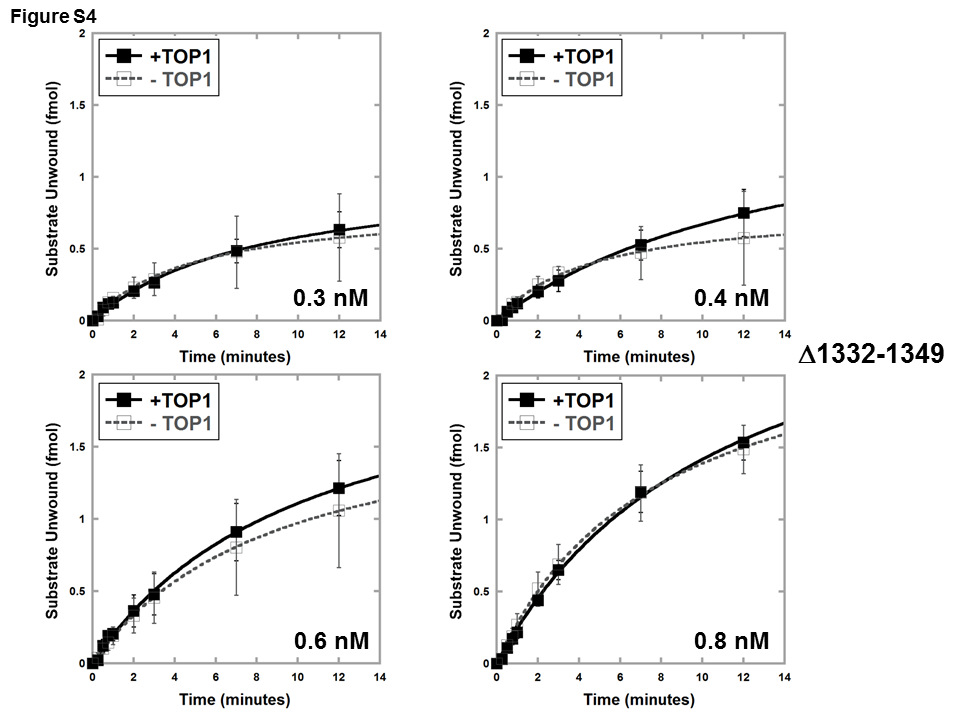


**Figure S4.** Unwinding of RNA:DNA substrate by BLM-Δ1332-1349 is not stimulated by TOP1. Changes in BLM-Δ1332-1349 activity with a RNA:DNA substrate by TOP1 was assessed by quantifying the unwinding as a function of time using a range of BLM protein concentrations with and without TOP1, as in Figure 2B. Curves were fitted to hyperbolic plots corresponding to the Michaelis-Menten equation.


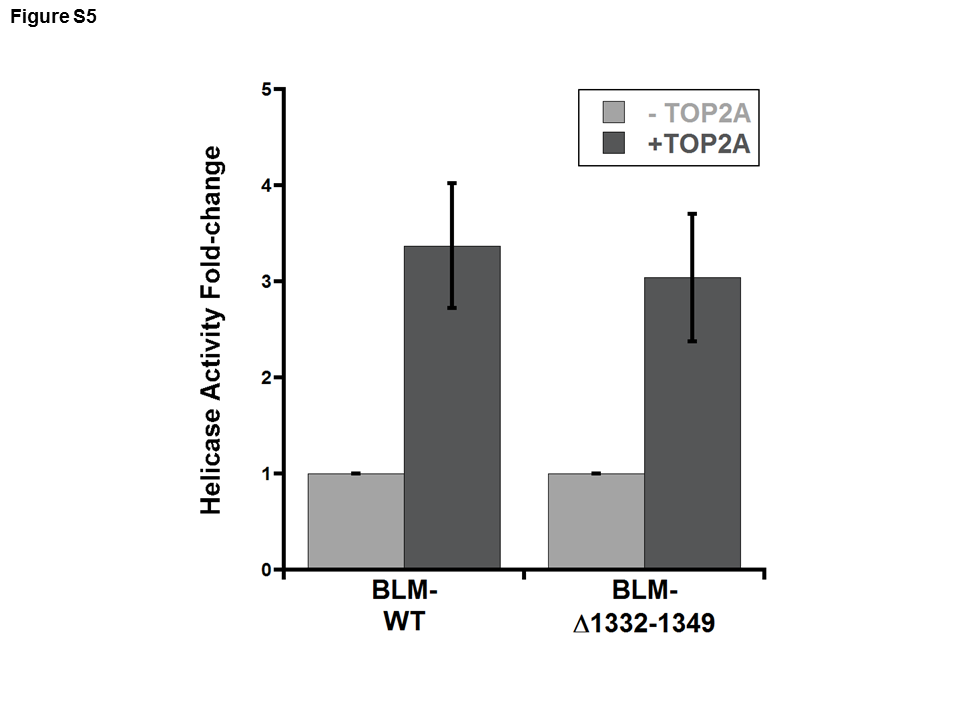


**Figure S5.** Unwinding of DNA:DNA substrate by BLM-Δ1332-1349 is stimulated by TOP2A. Unwinding of DNA:DNA substrate was assessed using protein concentrations yielding similar unwinding activities (0.08 nM BLM-WT or 0.13 nM BLM-Δ1332-1349), with 7 nM TOP2A. Reactions were stopped after 10 minutes, and double- and single-stranded products were separated on non-denaturing acrylamide gels. Unwinding was quantified as the amount of single-stranded substrate generated compared to the total substrate, and results represented as fold-change relative to minus TOP2A. Experiments were repeated 4 times. BLM-WT and BLM-Δ1332-1349 were similarly stimulated by TOP2A (ns, not significant; p>0.05). Error bars depict standard deviation. Statistical comparisons were made using a Student’s *t*-test.


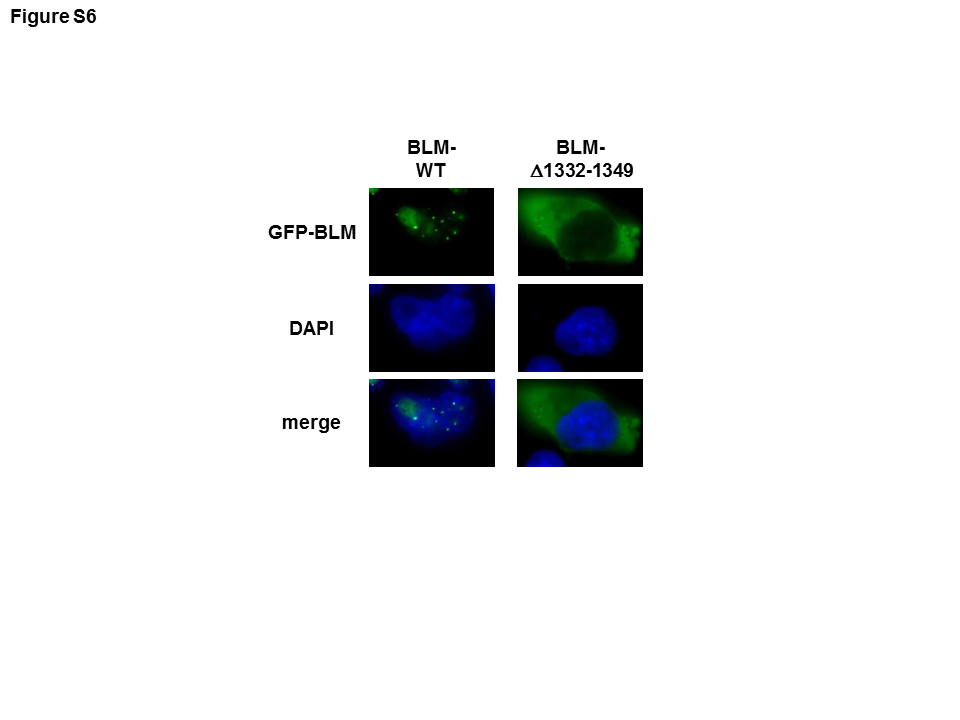


**Figure S6.** BLM-Δ1332-1349 does not localize to the nucleus. GM08505 *BLM*^-/-^ cells were transfected with the indicated GFP-tagged *BLM* plasmids, *BLM-WT* and *BLM-Δ1332-1349*, using Lipofectamine 2000. Cells were fixed with 4% paraformaldehyde 24-hours post-transfection, and coverslips mounted with VectaShield plus DAPI-mounting medium. The figure shows that while BLM-WT localizes to the nucleus, BLM-Δ1332-1349 localizes primarily outside the nucleus with an insignificant number of foci within the nucleus.


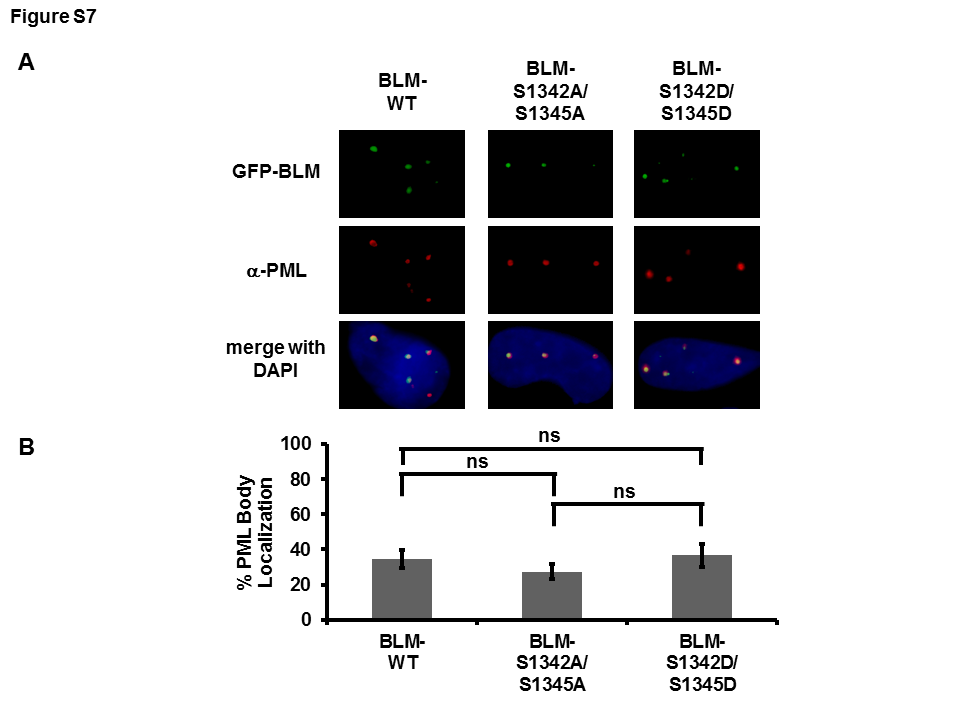


**Figure S7.** S1342 and S1345 do not affect PML body localization of BLM. (**A**) Cellular localization of BLM phospho-dead (BLM-S1342A/S1345A) and phospho-mimetic (BLM-S1342D/S1345D) mutants. GM08505 *BLM*^-/-^ cells were plated on sterile coverslips and transfected with the indicated GFP-tagged *BLM* plasmids, *BLM-WT*, *BLM-S1342A/S1345A* and *BLM-S1342D/S1345D*, using Lipofectamine 2000. Cells were fixed with 4% paraformaldehyde 24-hours post-transfection, permeabilized with 0.25% Triton-X-100 and blocked with 10% normal goat serum. PML bodies were stained with α-PML and Alexa-Fluor fluorescent secondary antibodies, and coverslips were mounted with VectaShield plus DAPI mounting medium. (**B**) Quantification of PML body localization. Co-localization of BLM and PML for 50 cells from 3 blinded experiments was expressed as percent PML body localization. All proteins had similar patterns of localization (ns, not significant; *p* > 0.05). Error bars depict standard deviation. Statistical comparisons were made using a Student’s *t*-test.


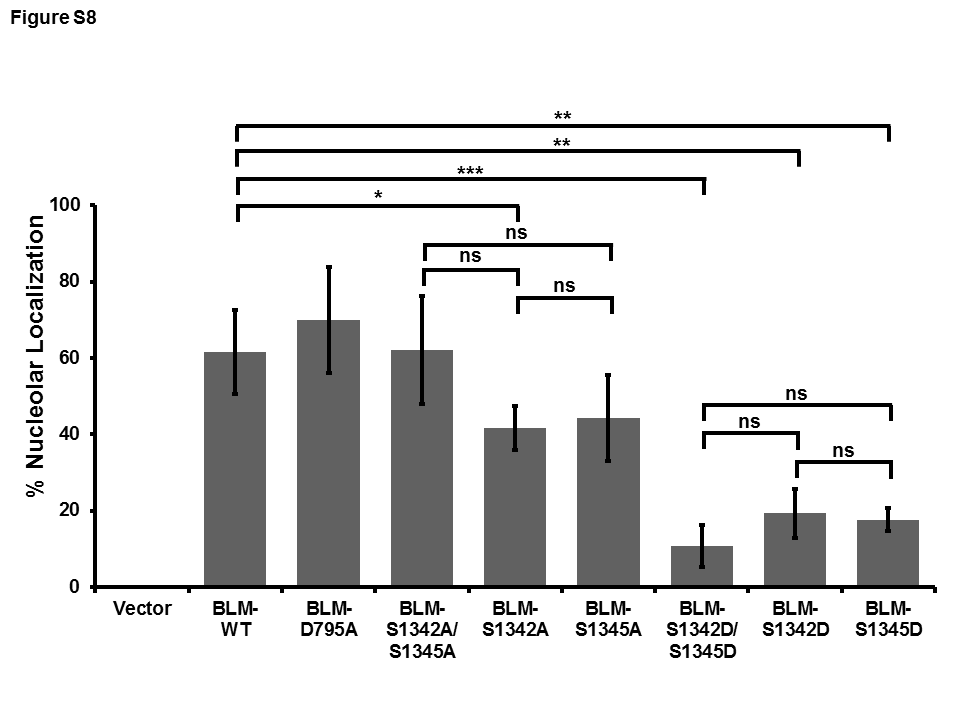


**Figure S8.** Nucleolar localization of BLM is affected by phospho-mimetic mutagenesis of BLM-S1342 and/or BLM-S1345. GM08505 *BLM*^-/-^ cells were transfected with the indicated GFP-tagged *BLM* plasmids, *BLM-WT*, *BLM-D795A* (helicase dead)*, BLM-S1342A/S1345A*, *BLM-S1342A, BLM-S1345A,* *BLM-S1342D/S1345D, BLM-S1342D,* *BLM-S1345D* and empty vector using Lipofectamine 2000. Nucleolar localization of BLM mutants for 100 cells from 3 to 5 blinded experiments was expressed as % nucleolar localization, as in Figure 4. BLM proteins with mutated S1342D, S1345D and S1342D/S1345D amino acids demonstrated significant differences in nucleolar localization compared to BLM WT, *p* < 0.001, *p* < 0.001 and *p* < 0.0001, respectively. However, single BLM mutants of either alanine or aspartate presented similar patterns of nucleolar localization to their cognate double mutants (ns, not significant; *p* > 0.05). Error bars depict standard deviation. Statistical comparisons were made using a using a Student’s *t*-test.


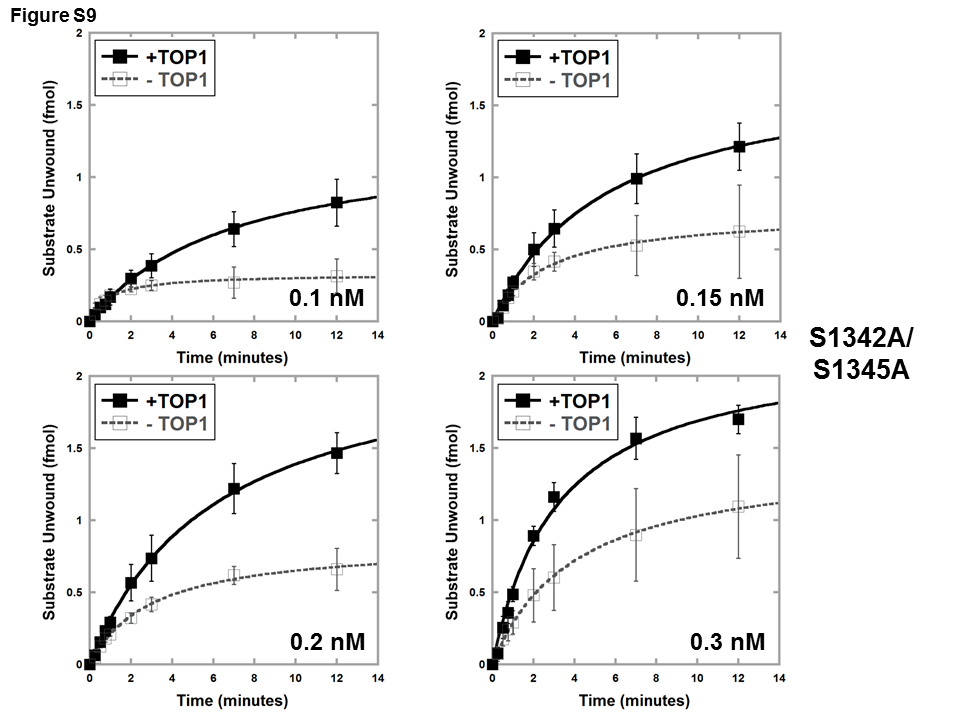


**Figure S9.** Unwinding of RNA:DNA substrate by BLM-S1342A/S1345A is stimulated by TOP1. Changes in BLM-S1342A/S1345A activity with a RNA:DNA substrate by TOP1 was assessed by quantifying the unwinding as a function of time using a range of BLM protein concentrations with and without TOP1, as in Figure 5A. Curves were fitted to hyperbolic plots corresponding to the Michaelis-Menten equation.


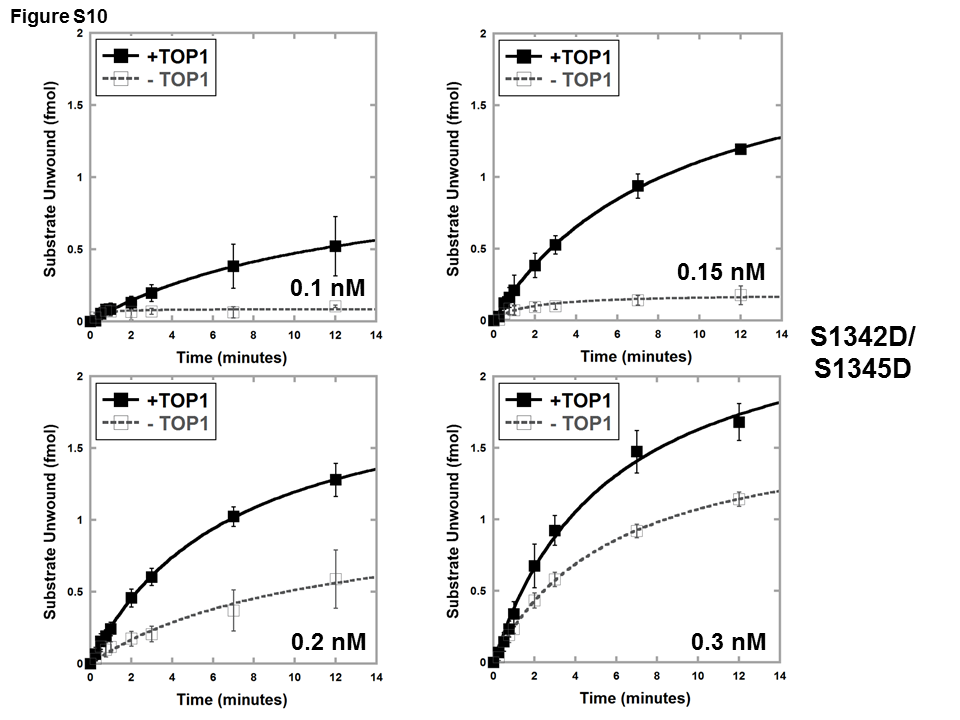


**Figure S10.** Unwinding of RNA:DNA substrate by BLM-S1342D/S1345D is stimulated by TOP1. Changes in BLM-S1342D/S1345D activity with a RNA:DNA substrate by TOP1 were assessed by quantifying the unwinding as a function of time using a range of BLM protein concentrations with and without TOP1, as in Figure 5B. Curves were fitted to hyperbolic plots corresponding to the Michaelis-Menten equation.
